# A Fog Computing and Cloudlet Based Augmented Reality System for the Industry 4.0 Shipyard

**DOI:** 10.3390/s18061798

**Published:** 2018-06-02

**Authors:** Tiago M. Fernández-Caramés, Paula Fraga-Lamas, Manuel Suárez-Albela, Miguel Vilar-Montesinos

**Affiliations:** 1 Unidade Mixta de Investigación Navantia-UDC, Universidade da Coruña, Edificio Talleres Tecnológicos, Mendizábal s/n, Ferrol 15403, Spain; m.albela@udc.es; 2 Navantia S. A., Astillero Ría de Ferrol, Taxonera, s/n, Ferrol 15403, Spain; mvilar@navantia.es

**Keywords:** augmented reality, Industry 4.0, shipyard, industrial augmented reality, Microsoft HoloLens, industrial operator support, fog computing, cloudlet, IIoT

## Abstract

Augmented Reality (AR) is one of the key technologies pointed out by Industry 4.0 as a tool for enhancing the next generation of automated and computerized factories. AR can also help shipbuilding operators, since they usually need to interact with information (e.g., product datasheets, instructions, maintenance procedures, quality control forms) that could be handled easily and more efficiently through AR devices. This is the reason why Navantia, one of the 10 largest shipbuilders in the world, is studying the application of AR (among other technologies) in different shipyard environments in a project called “Shipyard 4.0”. This article presents Navantia’s industrial AR (IAR) architecture, which is based on cloudlets and on the fog computing paradigm. Both technologies are ideal for supporting physically-distributed, low-latency and QoS-aware applications that decrease the network traffic and the computational load of traditional cloud computing systems. The proposed IAR communications architecture is evaluated in real-world scenarios with payload sizes according to demanding Microsoft HoloLens applications and when using a cloud, a cloudlet and a fog computing system. The results show that, in terms of response delay, the fog computing system is the fastest when transferring small payloads (less than 128 KB), while for larger file sizes, the cloudlet solution is faster than the others. Moreover, under high loads (with many concurrent IAR clients), the cloudlet in some cases is more than four times faster than the fog computing system in terms of response delay.

## 1. Introduction

Industry 4.0 is aimed at changing the way modern factories operate thanks to the application of some of the latest technologies associated with the Industrial Internet of Things (IIoT) [1], Big Data [2] or robotics [3]. Some Industry 4.0 technologies are already mature, but others are still being studied by manufacturers and academia for their application in industrial environments [4,5,6,7,8,9]. One of such technologies is Augmented Reality (AR), which, although it is based on different pioneering works performed in the 1960s [10,11], it was not until the late 1990s that it was actually pushed by academia [12] and by the industry (in this latter case, thanks to the support of the German government [13,14]). Since the 1990s, AR has been proposed to be used at different manufacturing stages [15], in assembly processes [16] or in nano-manufacturing [17]. The automotive industry has been the most active in the development of AR applications, creating solutions for the design phases [18,19] or for car assembly [20].

It is important to note that AR hardware and software have to include certain characteristics (e.g., ruggedness, battery life, robustness) to be used in industrial environments, which led to the concept of Industrial Augmented Reality (IAR). IAR devices usually have to receive data wirelessly and in real or quasi-real time in order to provide an attractive user experience. However, factories and other industrial environments are often a tough place for electro-magnetic wireless communications because of the presence of numerous and large metal objects that create reflections and even block signal propagation [21,22]. In addition, in an Industry 4.0 factory, IAR solutions are expected to provide dynamic on-demand information, which requires fast responses from remote data servers. Furthermore, such information may vary in payload size, because IAR devices can request from just a few kilobytes of text content to several megabytes of video. Therefore, IAR systems not only need to provide AR functionality, but also have to be able to exchange and manage content as fast as possible. In the past, researchers have addressed the high-detail 3D content transfer speed limitations mainly by trying to simplify or decimate such content [23,24,25], but although this can be useful in some cases, the loss of certain details in industrial designs may derive into problems (e.g., when using a simplified 3D design as a reference during manufacturing or in quality checks).

To tackle the previous issues, this article proposes the use of an IAR architecture based on the fog computing paradigm [26], which allows for supporting physically-distributed, low-latency and Quality of Service (QoS) aware applications that decrease the network traffic and the computational load of traditional cloud computing systems. In addition, note that fog gateways are usually constrained in terms of computing power [27], so, if an IAR system demands real-time rendering or compute-intensive services, other approaches, like cloudlets, are a better fit [28]. Therefore, the proposed architecture also includes a local cloudlet for performing the most demanding tasks. Thus, this paper presents the design, implementation and practical evaluation of such an IAR architecture. The following are the main contributions of the article, which, as of writing, have not been found together in the literature:

- It presents a novel architecture that merges cloudlets and fog computing devices to provide fast IAR device communications.
- It evaluates cloud, fog and cloudlet systems in real IAR scenarios when transferring payloads of different sizes.
- It also shows a performance evaluation in a scenario when up to 240 virtual IAR clients are transmitting payloads with a size according to demanding Microsoft HoloLens [29] applications.

The rest of this paper is structured as follows. Section 2 describes briefly the latest commercial and academic IAR systems, enumerates promising IAR applications for a shipyard and analyzes the most relevant IAR architectures. Section 3 presents the design and implementation of the IAR system proposed, while Section 4 studies the performance of the system in terms of latency response in different practical scenarios. Finally, Section 5 is devoted to conclusions.

## 2. State of the Art: AR in the Shipyard

### 2.1. Commercial and Academic Systems

There are not many well-documented AR and IAR solutions devised explicitly for shipbuilding environments. Regarding commercial systems, it is worth mentioning the work carried out by companies like BAE Systems, IndexAR or Newport News Shipbuilding. BAE Systems is currently developing IAR solutions [30,31] and has already tested virtual reality solutions at certain construction stages of the offshore patrol vessels and Type 26 frigates [32]. IndexAR [33] has developed diverse IAR applications for shipbuilding, mainly for easing construction, operation and maintenance processes. Similarly, Newport News Shipbuilding [34] developed an IAR system for enhancing safety, training, operation and maintenance processes performed in ships and shipyards.

In the academic field, although the first documented industrial developments date back to the early 1990s [35], just a few researchers have focused their work on developing novel AR and IAR solutions for shipbuilding. For instance, in [36] an augmented reality helmet is presented for welding that shows information, suggestions and possible errors on the tasks performed. Welding systems are also proposed in [37,38]. In [37] an IAR interface is described to control a welding robot in a ship under construction. In contrast, in [38] a training system is presented that makes use of a pair of smart glasses and a torch to simulate welding processes.

The use of IAR in design processes is popular in different industries, but there are not many examples for shipbuilding. An example is the work presented in [39], which describes an IAR solution for detecting differences between a design model and the real construction. In the case of finding a discrepancy, operators can notify it and then modify the CAD model or some elements of the construction to make them match.

Finally, it is important to note the possibilities that IAR can bring to maintenance tasks, which, traditionally, have been performed by using manuals that require previous instruction. Thus, systems like the one created by Havard et al. [40] allow for replacing traditional paper and electronic maintenance documents with a tablet that shows step-by-step instructions on the maintenance procedure.

### 2.2. Potential IAR Applications in a Shipyard

IAR can help shipbuilding processes in many scenarios. The most relevant are the following:

- Providing plant information. In the case of Navantia’s shipyards, most information about products and procedures is stored digitally, but operators still rely on paper documents.
- Performing quality controls. At the manufacturing stage most processes require a supervisor to perform a quality control where it is necessary to compare the design model with the actual product, which involves consulting datasheets and determining if the product requirements are fulfilled [41].
- Assisting operators during assembly and manufacturing processes. The manufacturing process of the products created in a shipyard (e.g., pipes, metal sheets) requires sequential steps that must be followed in a specific order. IAR can help operators by providing them with step-by-step instructions through intuitive interfaces, which have proved to reduce significantly the number of human errors [42].
- Locating items. Shipyard workshops are usually quite large, what makes it difficult to locate certain items. An IAR system can help operators by pointing at the specific location of the item or at the area where it is placed. In certain cases, this functionality may be implemented into the IAR software, but, in large spaces, it is more common to make use of sensors or third-party location systems. For example, in Navantia’s pipe workshop a pipe location system is deployed based on active Radio Frequency IDentification (RFID) tags [43,44,45] that can exchange data with IAR applications.
- Guiding and navigating environments. Shipyards are often quite large. In the case of Navantia’s shipyard in Ferrol (Galicia, Spain), it occupies 1,000,000 square meters. Therefore, it would be useful to guide operators through the optimal route when they go from one place to another [46]. Moreover, a guiding system may help indoors, in the workshops and warehouses, when looking for specific items or areas.
- Visualizing hidden areas. In many shipyard installations and inside a ship, part of the piping and wiring is installed behind walls or bulkheads, which makes it difficult to carry out maintenance procedures and to detect the origin of certain faults. An IAR display can superimpose digital 3D models with reality and then ease the location of the underlying infrastructure [47].
- System monitoring. IAR can work together with IIoT to show the information collected from sensors scattered throughout the shipyard [48]. Moreover, an IAR interface enables user-friendly and real-time interactions with certain objects and machines that behave as actuators [49].
- Performing predictive maintenance. The use of IIoT, Big Data techniques and IAR devices can help to detect and to warn operators about anomalies that may cause imminent or future problems [50].
- Augmented collaboration. In Navantia’s shipyards, like in other shipbuilding companies, communication systems usually only involve voice communications (e.g., walkie-talkies, mobile phones). IAR systems are able to provide first-person video and audio content to remote collaborators, which enriches the communication and eases remote guidance on the resolution of incidents [51]. In addition, IAR devices usually allow for creating enriched notes, enhancing dramatically traditional text-based reports [52].

### 2.3. IAR Architectures

IAR is a technology that projects digital information on displays, so it demands low-latency responses in order to create an attractive user experience. For this reason, the digital content is usually stored locally, first in permanent memory and then in RAM, which guarantees low data access times. Although such an approximation is fine for static content, an Industry 4.0 environment is characterized by being dynamic, especially when information collected from IIoT and third-party networks has to be displayed.

One strategy to load information dynamically is caching: when the IAR solution predicts that there is a high likelihood that certain information will be required, an IAR device can download through a background process such content. However, note that when using local caching, algorithms become more complex, since the prediction algorithm has to be effective and it has to be checked periodically if the memory contains the latest version of the data. Moreover, cache synchronization becomes expensive in terms of energy cost in the case of exchanging large files that may end up not being used.

Because of the previously mentioned limitations, researchers have modified traditional AR architectures. The most common variation consists in adding local PCs to the network in order to increase computational capabilities. For instance, an interesting work is described in [53], where it is studied how to deploy AR systems in vehicles. Although the researchers state that it is easy to create centralized in-vehicle AR systems when relying on traditional Engine Control Units (ECUs), such an approach derives into an increasing response latency due to the multiple tasks to be carried out (e.g., image acquisition and display, protocol conversions, sensor value collection and processing). Moreover, the researchers indicate that the latest trends in car manufacturing regarding vehicle architecture design prefer to go for architectures divided into domains (for instance, there might be a domain for the powertrain, one for the transmission and another one for chassis control), among which an automotive AR system would distribute its functionality.

The use of remote processing servers has also been proposed, which led to central server-based and cloud computing-based architectures. An example of such a kind of architecture is detailed in [54]. In such a paper, an AR solution is presented based on a five-layer architecture that focuses most of the processing in a central server, which exchanges data through HTTP while relying on web services.

The latest trend in processing for AR solutions consists in the use of edge computing. Note that such a paradigm is really close to fog computing and, in fact, some authors consider fog computing as one of the possible implementations of edge computing, together with cloudlets and mobile edge computing [28]. An example of an AR application that uses edge computing is presented in [55]. There, the authors emphasize the fact that current mobile AR devices still have to progress in terms of computational and graphical performance, being essential to decrease end-to-end latency, especially for video transmissions. To tackle such issues, the researchers propose an AR edge computing architecture to offload the demanding real-time AR algorithms to a high-end PC placed in the local network. During the experiments, the authors measured an end-to-end latency of roughly 50 ms when transmitting compressed video frames, which they consider acceptable for handheld AR devices, but too high for most Head-Mounted Display (HMD) solutions. Moreover, the researchers conclude that nowadays the transmission of raw video data still requires communication interfaces with higher bit rates and smaller latencies.

## 3. System Design and Implementation

### 3.1. Communications Architecture

**Figure 1:**
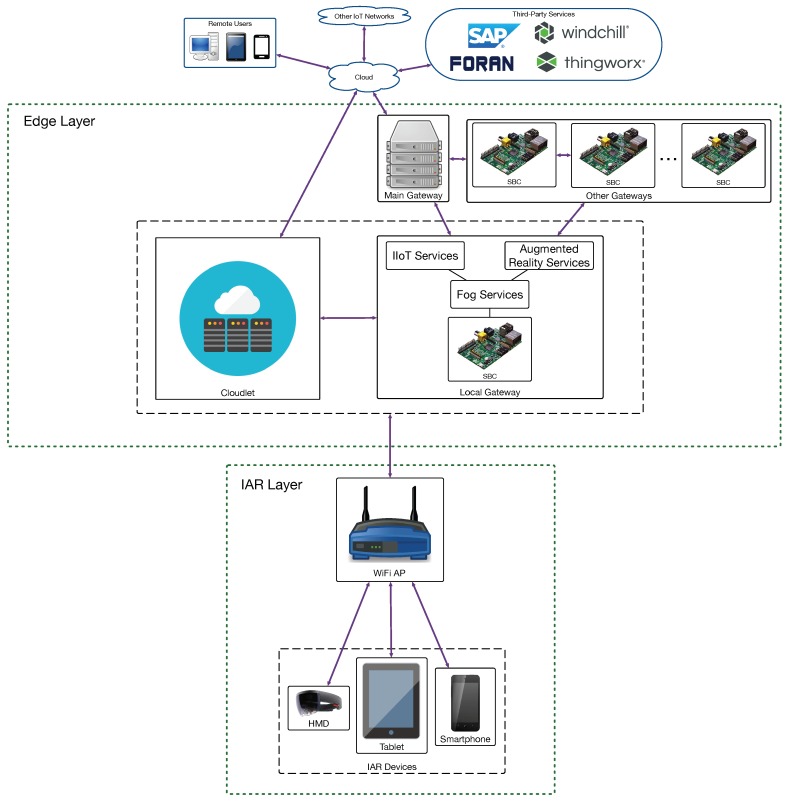
Proposed IAR communications architecture.

After analyzing the state of the art and the requirements of potential shipbuilding IAR applications, the communications architecture was proposed, as shown in Figure 1, which is a three-layer edge computing architecture. The layer at the bottom includes the IAR devices, which make use of WiFi connectivity since Navantia’s shipyard in Ferrol has already deployed IEEE 802.11 b/g/n/ac infrastructure and because it is the most popular medium-range wireless standard used by tablets, smartphones and smart glasses [56]. Nonetheless, the architecture can make use of other wireless technologies.

Each IAR device exchanges data with a local Edge Layer gateway, which is usually the one that is physically closer. All the local gateways are part of a fog computing sub-layer that provides different low-latency services, which include, in the case of the proposed IAR system, sensor fusion and data caching for sending content to the IAR devices. Although most of the time only one gateway provides fog services to a specific IAR device, the gateways of the Fog Layer can cooperate to offer complex services, to distribute compute-intensive tasks and to ease the collaboration between remote IAR devices. In Figure 1 every local gateway is represented by a Single-Board Computer (SBC), which is a reduced-size low-cost computer that can be installed easily in any shipyard workshop. Thus, the architecture assumes that SBCs that act as gateways are scattered throughout the shipyard.

In addition, the Edge Layer contains cloudlets, which are able to carry out compute-intensive tasks like rendering or image processing. Since a cloudlet is really close to the IAR devices that request its services, their response latency is lower than the one offered by traditional cloud computing systems.

Finally, at the top of Figure 1 is Navantia’s cloud, where the services that require more processing power are executed. The cloud also connects with third-party systems that are part of Navantia’s IT core. Specifically, software from SAP [57] is used as Enterprise Resource Planning (ERP) and Manufacturing Execution System (MES), PTC’s Windchill [58] is the Product Life-cycle Management (PLM) software, shipbuilding models are created with FORAN [59], and ThingWorx [60] is being tested as an IIoT platform.

### 3.2. IAR Software and Hardware

Both software and hardware are key in IAR applications [48]. Regarding the hardware, it is possible to use regular smartphones and tablets, although, for industrial environments, they should be protected against dirt, dents and drops. The evolution of the technology in the last years has led the manufacturers to develop solutions embedded into smart glasses [61,62,63], helmets [29,64] or HMD devices [65], which are really promising, but that are still expensive (between US $500 and $5,000) and can be considered experimental in terms of features for industrial environments. Such ideal features include: being as light as possible, the battery should last through the whole working day, the field of view should be as wide as possible, and devices should embed voice-recognition features to enable hands-free operation.

With respect to the software, there are many AR Software Development Kits (SDKs) that can be used for developing IAR implementations, like ARToolkit [66], Mixare [67], Vuforia [68] or Wikitude [69]. An SDK should include the following features to be able to create successful IAR applications [15]:

- It should be as accurate as possible, reliable and provide fall-back alternatives.
- An IAR application should be user-friendly, making as easy as possible its setup and the learning period.
- The application should be scalable, since factories involve many users interacting concurrently. The advantages and disadvantages of cloud and fog computing architectures have to be weighted in order to determine which fits better depending on the user requirements.

For the tests performed in this article, among the different hardware devices available, Microsoft HoloLens smartglasses were selected. Although they are not cheap (around US $3000 as of writing), they are one the most promising AR headsets on the market thanks to its power (it contains a custom Holographic Processing Unit (HPU), 2 GB of RAM and 64 GB of on-board storage), embedded sensors, display (it includes see-through holographic lenses, automatic pupillary distance calibration, 2.3 million light points of holographic resolution) and potential interfaces (voice commands, gaze tracking and gesture recognition). In addition, the smartglasses can be connected to other devices through WiFi (IEEE 802.11 ac) or Bluetooth 4.1, and its battery lasts approximately 2 to 3 h with intensive use (two weeks in stand-by).

Furthermore, It should be mentioned that the HoloLens tracking system operates in a different way with respect to traditional marker-based AR systems [56], since the smartglasses make use of two visible-light black and white cameras that observe the features of the environment. Once such features are determined, the smartglasses fuse the information about them with the Inertial Measurement Unit (IMU) data in order to locate the user within the environment. Therefore, video tracking is performed entirely by the HoloLens so the developer does not have to worry about the position of the virtual markers, but only about the virtual content to be displayed.

## 4. Experiments

### 4.1. Experimental Setup

**Figure 2:**
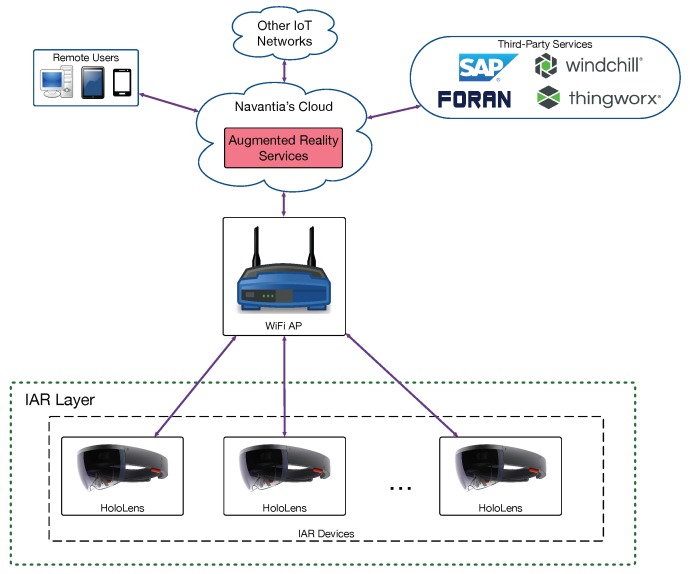
Implemented cloud-based architecture.

**Figure 3:**
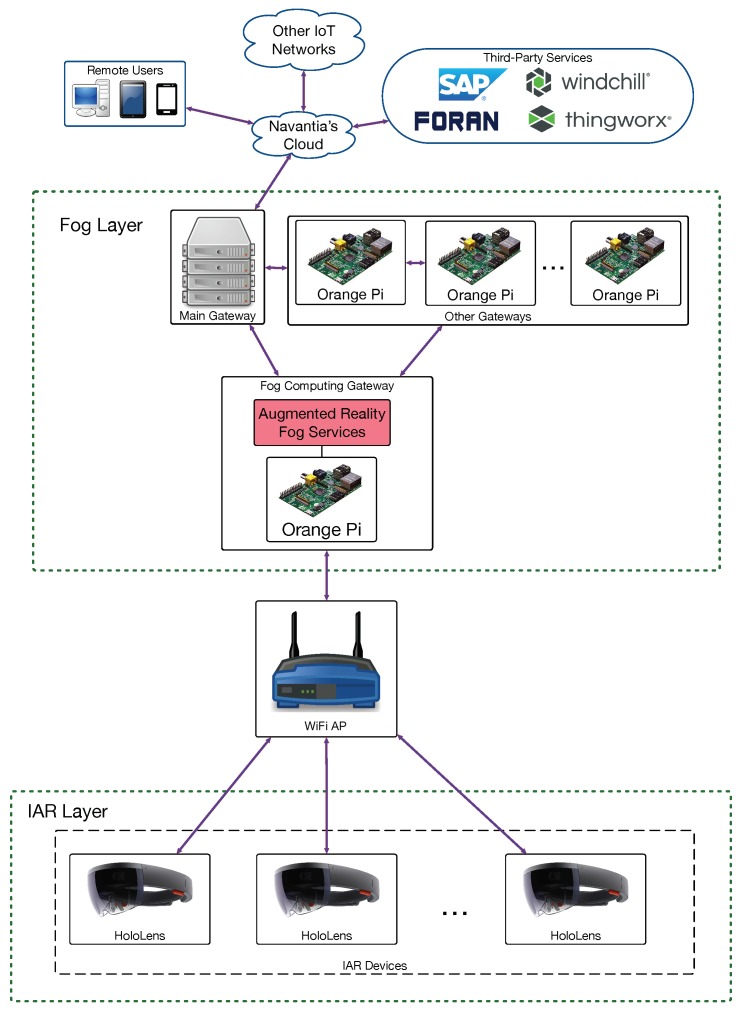
Implemented fog computing-based architecture.

**Figure 4:**
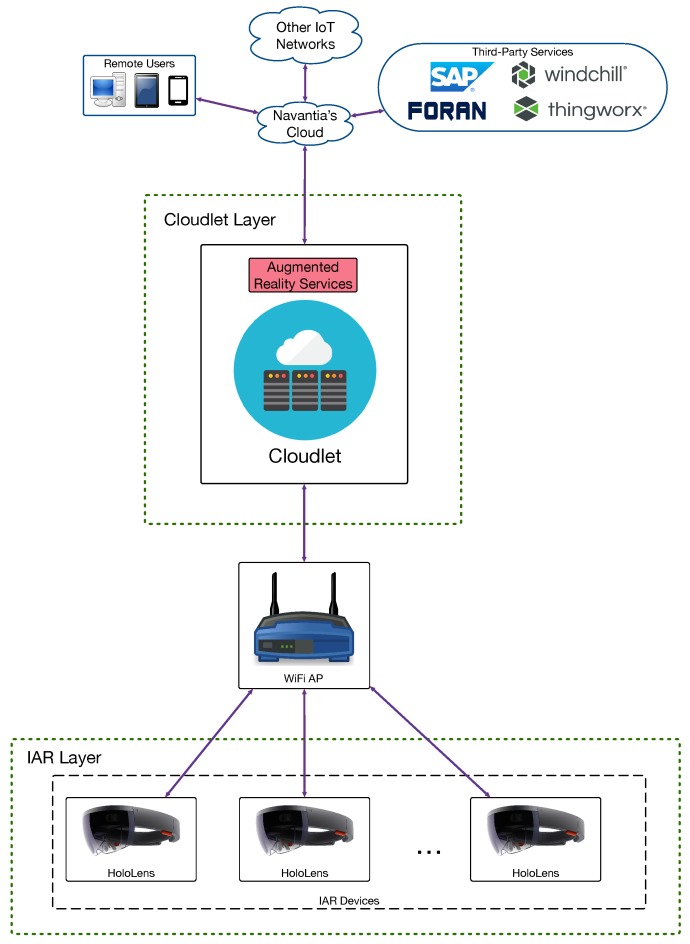
Implemented cloudlet-based architecture.

In order to determine the performance of the proposed architecture in terms of response delay, three scenarios were evaluated depending on where the augmented reality services were provided. This resulted in the three architectures depicted in Figure 2, Figure 3 and Figure 4 (for cloud, fog computing and cloudlet-based architectures). Each architecture contains a subset of the components of the edge-computing architecture previously illustrated in Figure 1. In the case of Figure 2, the different IAR devices are connected through WiFi access points to Navantia’s cloud, which runs the augmented reality services. In contrast, in Figure 3, such services are provided by the different SBCs of the fog layer. In Figure 4, a cloudlet is the device responsible for executing the mentioned services.

**Figure 5:**
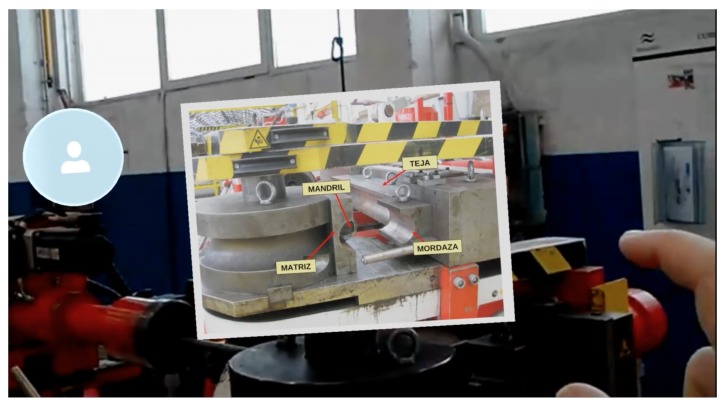
Microsoft HoloLens collaborative IAR application.

**Figure 6:**
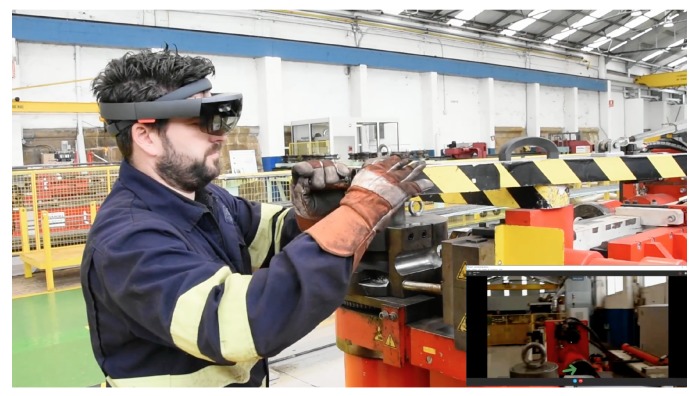
One of the tests in the pipe workshop with the HoloLens augmented collaboration application.

**Figure 7:**
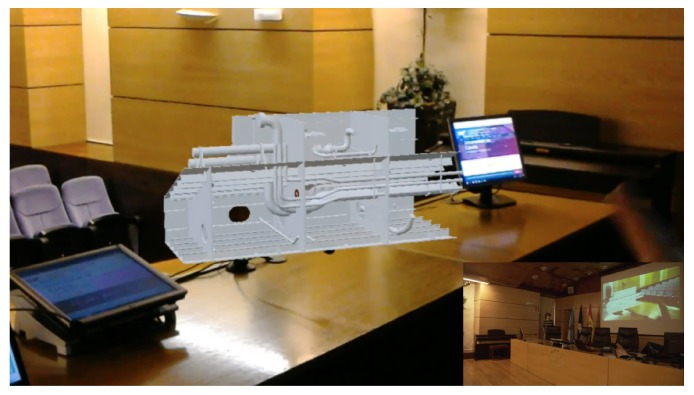
Ship block projected during a meeting.

Before carrying out the experiments, the transfer speed requirements of the system when running wirelessly were determined, at a medium distance (roughly three meters from the IAR server); there are two representative IAR applications on the Microsoft HoloLens smartglasses:

- A Holo app (an application for HoloLens) that embedded Skype for remote augmented collaboration in the shipyard’s pipe workshop. Figure 5 shows a screenshot of the application. In the Figure, one of the moments when a remote worker (who is using Skype) guides the operator wearing the Microsoft HoloLens through the steps for performing a specific maintenance procedure on a bending machine is shown. Figure 6 shows the mentioned operator following the remote instructions. The data exchanged by the HoloLens with the upper layers of the architecture were basically Skype frames, which make use of a closed-source protocol that is able to stream audio (voice-over-IP from the HoloLens towards the remote collaborator and from such a collaborator towards the operator with the HoloLens) and video (it was only streamed video from the HoloLens front-facing camera to the remote collaborator).
- The second Holo app was a streaming application that projected highly polygonal models. Such models were extracted from FORAN in JT and OBJ formats. Since the total file size of the raw 3D models was actually large (1.5 GB), before including them in the Holo app, they were polished with Blender [70] to reduce the number of required triangles (the raw 3D models were made of 630,477 triangles). The final models only use 65 MB of disk and 28,726 triangles. Figure 7 shows a screenshot of the app when projecting a ship block during a design meeting. The models were first downloaded into the HoloLens and then they were projected in a specific place (selected by the user) of the meeting room. The mixed reality 720p video captured by the HoloLens was then sent via WiFi through a streaming service to a remote computer that was connected to an HDMI projector.

For both tested applications, the exchanged data were sniffed using WireShark and the average data transfer speeds were determined. In the case of the Skype-embedded Holo app it was 8780 KB/s, while the video streaming app averaged 369 KB/s. It is also interesting to identify the type and amount of the exchanged data. In the case of the Skype-embedded Holo app, when performing a maintenance operation in the bending machine that took 632 s, 28,643 packets were exchanged between a remote PC and the HoloLens, transferring a total of 9.49 MB. Most of this traffic (98%) was related to compressed audio/video data, being that the rest of the packets were associated with network signaling and control protocols. A similar ratio between data and control frames was obtained by the video streaming Holo app, but the amount of exchanged data depended heavily on the complexity of the exchanged 3D models.

Then, in order to automate the tests and to be able to increase the number of concurrent IAR devices, a laptop (Intel i7-5500U@2.4 GHz processor, 250 GB SSD, 8 GB of RAM and a Gigabit Ethernet connection) with a virtual machine (64-bit Debian 9) was used to carry out IAR file exchanges. To limit the performance of the laptop when simulating the use of a true IAR device, the virtual machine was configured to use only two cores and 2 GB of RAM. Note that a laptop was selected due to practical reasons and because what was measured was the latency response, but the measurement methodology and conclusions would be identical when performed through the actual smartglasses. However, it is worth mentioning that all the communications performed by the Holo apps are exclusively wireless, while the laptop allows for using an Ethernet connection, thus avoiding the impact on the latency response of the wireless scenario (e.g., distance to access points, obstacles, presence of metal) and therefore providing a fairer evaluation of the different architectures.

In the case of the fog computing scenario, an Orange Pi SBC was used. Such a device integrates a low power-consumption SoC running a quad-core ARM Cortex-A7 processor at 1.6 GHz, 1 GB of RAM, a 10/100 Mbit Ethernet interface and a micro-SD card slot. The cloudlet consisted of a 64-bit Debian 9 virtual machine deployed on a Dell PowerEdge R415 local server configured with one core (AMD Opteron@3.1 GHz), 2 GB of RAM and a Gigabit network interface. In these two scenarios, the exchanges were always performed inside the local network of the shipyard, without needing to reach the Internet. In such a local network, under regular network traffic conditions, the minimum/average/maximum round-trip times to the cloudlet were 0.935/1.034/1.695 ms, while the same times were 0.895/0.977/1.074 ms for the local fog gateway. Finally, in a third scenario, the performance of the cloud computing architecture was evaluated, which, for the sake of fairness, made use of the same server as the cloudlet, but it was deployed in a remote Internet location with minimum/average/maximum round-trip times of 47.25/49.3/62.87 ms.

The software for the experiments was configured exactly in the same way for all the tests. In the server-side, four concurrent processes waited for requests. At the client-side 20 concurrent clients were simulated. The clients asked for files (i.e., plain data or multimedia objects) that were required by the local IAR software. Such a number of clients was selected for the sake of fairness when comparing the different architectures, due to the hardware constraints of the fog gateways. Therefore, it was assumed that, in a real deployment, in the physical area covered by a fog gateway in a shipyard, there would be up to 20 workers using IAR devices simultaneously. In such scenarios, every exchanged file, whose size varied from 32 B to 8 MB, was downloaded 1000 times.

### 4.2. Response Latency Comparison

One of the main challenges of IAR deployments is that IAR devices should be able to obtain and display the required information fast enough to be presented seamlessly to the user. This speed limitation is determined by different factors (e.g., involved hardware, communication technology, evaluated scenario), but, during the experiments, most of them were fixed, thus reducing their impact on the obtained absolute results. The only exception was the network traffic, since the experiments were carried out on local and Internet-connected networks shared with other users (their influence can be decreased by averaging many tests). Therefore, in this subsection, the performance of the proposed fog, cloud and cloudlet-based architectures was evaluated in order to determine which should be used when exchanging IAR payloads of different sizes.

**Figure 8:**
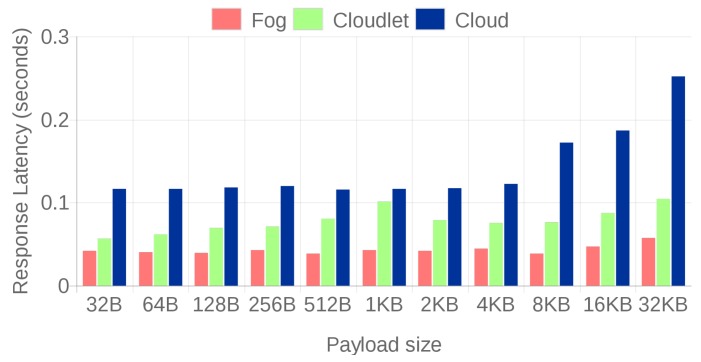
Response latencies for 32 B to 32 KB file sizes.

**Figure 9:**
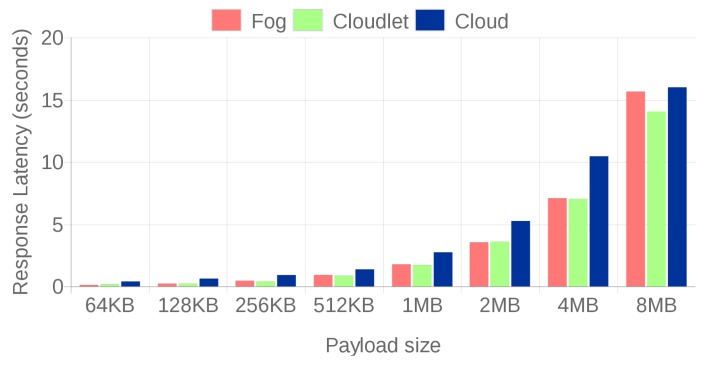
Response latencies for 64 KB to 8 MB file sizes.

Figure 8 and Figure 9 compare the response latencies for the three scenarios. For the sake of clarity, the results were split into two different figures, distinguishing between small file sizes (Figure 8) and large file sizes (Figure 9). Small files are mainly related to certain contextual data (for instance, characteristics of the pipes obtained from SAP), while large files would be associated with video streaming of design models from FORAN.

Before drawing conclusions from the Figures, it is important to note that, for the largest files (4 and 8 MB), Table 1 must be taken into account. Such a table shows the percentage of 4 and 8 MB files that were transmitted successfully. While for other file sizes there was a 100% success rate, for the largest sizes, the rate dropped remarkably in fog and cloud computing scenarios due to the joint effect of file size, the internal processing lag (in the case of the fog gateways) and communication delays (that mainly affect the cloud). Therefore, as a result, Figure 8 and Figure 9 show the average results for the successfully transmitted files.

In addition, note that slight fluctuations can be observed in the Figures due to the network load, but they do not alter the conclusions.

For small file sizes (up to 128 KB), fog gateways respond faster, despite their hardware constraints, thanks to their low communication latency. Moreover, it can be observed that cloud-based communications are clearly slower. However, in the case of large files (more than 128 KB), the cloudlet architecture is the one that obtains the smallest latencies, both thanks to its proximity to the IAR devices and to its computing power, which is superior to the one of a fog SBC. Nonetheless, the latency differences between the fog computing and the cloudlet-based systems may not seem as large as expected when taking into account their difference in power and cost. In spite of the lack of such differences, what makes the cloudlet system a clear winner for large file exchanges is that, as it can be observed in Table 1, it is the only system capable of providing a 100% transmission success rate.

### 4.3. Sample Processing Rates under High Loads

The previous tests allowed for establishing a performance baseline for fog and cloudlet scenarios and for discarding a cloud deployment, since it presents excessive response latency. To compare in a fair IAR scenario the performance of the proposed fog and cloudlet architectures, a new set of tests was carried out by taking into account the actual Microsoft HoloLens data transfer speeds for the two Holo apps previously described in Section 4.1. The methodology used for obtaining such transfer speeds was also applied to the scenarios evaluated in the previous section and it was concluded that the closest values to the HoloLens data transfer rates (8780 B/s for the Skype-embedded Holo app and 369 KB /s for video streaming) were achieved when downloading the 64 KB and the 32 B files, which obtained practical data transfer rates of 392.45 KB/s and 13.15 KB/s, respectively. With these two file sizes, a total of 50,000 file transfers were performed for different concurrency levels: from 30 to 240 simultaneous clients. To cope with such a large number of concurrent connections, a virtual machine that had four cores and 8 GB of RAM that was hosted on a server with an Intel Core i7-4790 @ 3.6 GHz, 32 GB of RAM and a 500 GB SSD was used.

**Figure 10:**
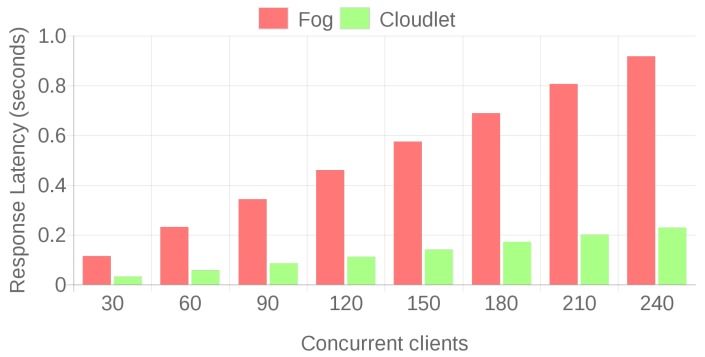
Latencies for 32 B and 30 to 240 concurrent clients.

**Figure 11:**
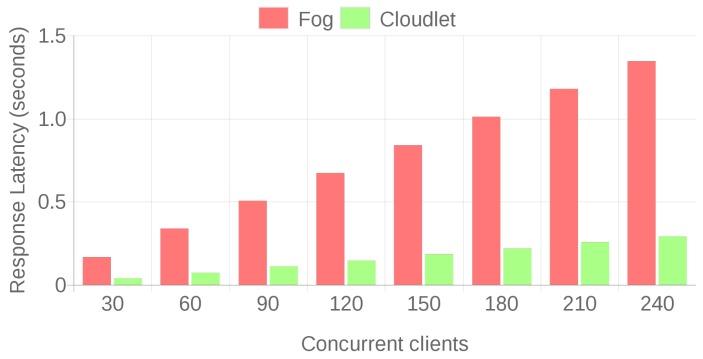
Latencies for 64 KB and 30 to 240 concurrent clients.

The obtained results are presented in Figure 10 and Figure 11. As it can be observed, when a large number of concurrent users requests data from the server, the fog deployment rapidly falls behind the cloudlet in terms of response latency and throughput. In the case of the largest number of concurrent clients, the response latency of the fog computing based system is four times greater than the one of the cloudlet for both 32 B and 64 KB file sizes. Regarding successful transaction rates, both the cloudlet and the fog deployments reach 100% for all the tests, but, at the view of the response latencies, it is clear that, in high-load scenarios, the cloudlet outperforms the fog computing system.

### 4.4. Key Findings

After analyzing the results of the tests described in the previous subsections, the following conclusions can be drawn:

- When a single IAR device operates in a specific shipyard area, for files of up to 128 KB, fog gateways respond faster despite being less powerful, cheaper and less energy demanding than a cloud or than the typical high-end PC used by a cloudlet.
- In the case of exchanging large files, the cloudlet-based solution is the fastest of the three tested architectures. However, in some scenarios it might be less expensive to make use of fog computing and SBCs, which are slightly slower, but cheaper in terms of acquisition cost and energy consumption.
- When a relevant number of IAR devices transmit concurrently, a cloudlet responds faster than a remote cloud or a fog gateway.

In addition, it must be emphasized that, despite the previously mentioned results, in a real environment, several types of IAR data and applications are involved, so the ideal architecture would consist in the combination of the three alternatives that conform to the proposed edge computing architecture. In this way, an IAR device would have to choose in real time the data source that would optimize the user experience. In addition, for every specific scenario it should be considered whether the content can be previously simplified in terms of detail in order to decrease the amount of data transferred to the operator’s IAR device.

Furthermore, at the view of the obtained results, it can be concluded that the proposed communications architecture (previously depicted in Figure 1) may be scaled to the whole shipyard by adding infrastructure to every area where IAR devices need to be used. Working areas differ in size depending on the workshop and on the tasks to be performed, but for the currently planned needs of the evaluated shipyard (in terms of potential IAR devices that are planned to operate concurrently), a cloudlet would be enough for every workshop and a fog computing gateway would be required for covering a working area of 1000 square meters. However, note that the scale of the infrastructure is highly dependent on the number of potential IAR devices operating in the workshop (in the case of cloudlets) or in a specific area (when determining the number of fog computing gateways).

Finally, it is worth indicating the advantages and disadvantages collected from the operators after testing the Holo apps. First, it must be pointed out that all workers were really impressed by the augmented reality experience provided by HoloLens. In fact, the feeling of having an immersive experience was unanimous. Under a regular use, no relevant lag was experienced, except for certain moments when the WiFi connection was dropped or when large image files were transferred to the operator that wore the HoloLens.

After all the practical tests, the plant operators found the remote collaboration application useful. However, while the Holo app that projects ship 3D models made a great impression on Navantia’s designers, plant operators consider that such an app would not be used frequently in their everyday tasks.

In addition, it has to be mentioned that, after carrying out experiments with both applications at different workshops of the shipyard, even with the presence of operators performing welding tasks or using other machinery and tools, reliability issues (e.g., object tracking problems) were not observed. This can be mainly attributed to the feature extraction algorithm based on two black and white cameras and to the use of IMU data to estimate pose continuously. Note that, in contrast, other traditional AR systems based on markers are heavily influenced by lighting and by the characteristics of the camera, so their performance falls dramatically under low light conditions or when there is a high contrast [56].

Despite all the benefits of the proposed system, several issues arose:

- Although, in general, the smartglasses were easy to adjust to the worker head, in some specific cases it took time to find the right position to align the operator’s sight with the display.
- In certain areas of the shipyard where helmets were required, it was difficult for the operator to wear both the HoloLens and the safety helmet at the same time. Therefore, future IAR devices should take safety procedures into account.
- Since virtual and real objects live together under the operators point of view, it is possible that certain virtual objects may hide relevant events of the real world. Although operators can move most virtual objects around the working environment, occlusions have to be prevented as much as possible in order to avoid possible accidents.
- Although the operators did not complain about the comfort of the smartglasses, after using them for a certain time (around an hour), the users had use marks on their noses due to pressure. There were no complaints about headaches, nausea or loss of visual acuity.
- The system was not tested during a whole working shift (around eight hours), but at certain production stages for less than two hours in total, since that is how long the batteries lasted. Therefore, future IAR devices would have to extend battery life, while preserving a light weight.
- Despite HoloLens’ excellent performance for tracking objects, note that their memory is limited, which implies that they can only map a limited area. Such an area is related to the size of the triangle mesh created by the smartglasses. In the experiments performed inside large workshops, the mesh reached roughly 600,000 triangles and the tested apps kept on working flawlessly. However, it is important to emphasize that the number of triangles depends highly on the scenario, so a large and complex environment like a shipyard cannot be mapped directly into the HoloLens’ memory. Nonetheless, it is worth noting that most shipyard operators work in very specific areas (i.e., their work is performed in a certain area of a workshop) and that, in the case of requiring to map large areas, it is possible to make use of spatial-mapping mesh-caching techniques to load content dynamically from the fog computing or cloud system depending on the detected area.

## 5. Conclusions

IAR can be a really useful technology for the Industry 4.0 shipyard, but current architectures still have to be optimized and analyzed to adapt to the fast responses required by certain dynamic IAR applications. In this article Navantia’s IAR architecture has been described, which makes use of cloudlets and fog computing to reduce latency response and offload traditional cloud-based systems. After detailing the proposed architecture, it was evaluated in different real scenarios and for diverse payload sizes, concluding that, in terms of response delay, fog gateways are the fastest when transferring small payloads, but a cloudlet is faster for medium and large IAR payloads. In addition, when many IAR clients access concurrently the IAR service, the cloudlet is also remarkably faster than the fog computing system (in some cases more than four times faster). In any case, the proposed architecture is able to provide low-latency responses by mixing elements of both computing paradigms that may enable the development of future real-time IAR applications for the Industry 4.0 shipyard.

## Figures and Tables

**Table 1:** Successful transmissions of 4 MB and 8 MB files.

Approach/File Size	4 MB	8 MB
Cloudlet	100%	100%
Fog	100%	47.8%
Cloud	96.5%	32.1%

## Abbreviations

The following abbreviations are used in this manuscript:

AR	Augmented Reality
ERP	Enterprise Resource Planning
HMD	Head-Mounted Display
IAR	Industrial Augmented Reality
IIoT	Industrial Internet of Things
IMU	Inertial Measurement Unit
MES	Manufacturing Execution System
PLM	Product Life-cycle Management
RFID	Radio Frequency IDentification
SBC	Single Board Computer
SoC	System-on-Chip
